# Supplementary material for: A discordance of seasonally covarying cues uncovers misregulated phenotypes in the heterophyllous pitcher plant Cephalotus follicularis
Source: Proc Biol Sci. 2021 Jan 27;288(1943):20202568. doi: 10.1098/rspb.2020.2568 (PMC7893253; doi:10.1098/rspb.2020.2568)
Supplement: Supplementary Information [file rspb20202568supp1.pdf]

Supplementary Materials for

A discordance of seasonally covarying cues uncovers misregulated phenotypes in the heterophyllous pitcher plant *Cephalotus follicularis*

Deleted: unmasks a hidden reaction norm

Kenji Fukushima, Hideki Narukawa, Gergo Palfalvi, and Mitsuyasu Hasebe

List of Supplementary Materials:

Supplementary Texts 1–2
Supplementary Methods
Figs. S1–S12
Tables S1–S3 (separate file)
Supplementary Data (available on FigShare)
Supplementary References

Deleted: 1

Supplementary Texts

Supplementary Text 1. The effect of daytime/nighttime temperature in relation to the dual-cue responses. Given the shared sensing mechanisms for temperature and light in plants, it may not be surprising that both influence the heterophylly of *Cephalotus*. In *Arabidopsis thaliana*, phytochrome B (PHYB), a well-characterized photoreceptor, senses light by changing its conformation from the biologically inactive form (P_r) to the active form (P_{fr}) upon red light absorption [1]. The P_{fr}-to-P_r reversion can be driven not only by far-red light absorption, but also by high ambient temperatures (i.e., thermal reversion); therefore, PHYB serves as a sensor for both temperature and light [2,3]. Although other sensor proteins and downstream signaling components may enable the separate processing of the two cues, their sensing is not completely independent. The P_{fr} form of PHYB changes the activity of a developmentally important basic helix-loop-helix transcription factor PHYTOCHROME INTERACTING FACTOR4 (PIF4), thereby modulating morphogenetic responses such as shade avoidance and thermomorphogenesis [4]. Because the activities of PIF4 and other related proteins are controlled by circadian clock components [5–8], the morphogenetic responses are subjected to circadian gating, which refers to the differential responses conditional to the time of day [9].

Because circadian gating could affect heterophylly, we subjected *Cephalotus* to distinct daytime and nighttime temperature regimes (25°C and 15°C, respectively, or the opposite) in combination with three photoperiods (8L16D, 16L8D, and 24L0D) for 12 weeks. The heterophylly responses using calculated mean temperatures were largely compatible with those observed under constant temperatures (Fig. S4; Fig. 4C), suggesting that the use of constant temperatures is a reasonable approximation of daily fluctuating temperatures in analyzing the heterophylly of *Cephalotus*. Pitcher leaves were produced at a high proportion when the plants were exposed to 25°C in the dark period of long-day conditions (Fig. S4), even though the calculated mean daily temperature was relatively low (18.3°C). Although the effect might not be substantial under the conditions we tested, this result implies the potential involvement of circadian gating in modulating the heterophylly of *Cephalotus*.

Supplementary Text 2. The regional correlation of environmental factors in relation to water availability. Among the environmental factors other than temperature and photoperiod, water availability is of particular interest in relation to the cost-benefit model of plant carnivory, which predicts water and light availabilities as key factors in the phenotypic plasticity of carnivorous plants [10,11]. A typical habitat of *Cephalotus* is a peat-soil swamp in permanently damp seepage areas [12], and therefore water is available throughout the year without limitation. In such an environment, photoperiod tends to correlate positively with the photosynthetic benefit resulting from carnivory [10,11]. However, the winter-rainfall climate of the south-western corner of Western Australia inverses such relationships outside the water-rich regions (*i.e.*, negative correlation between photoperiod and water availability). This fact highlights an attractive hypothesis that the breakdown of a regional environmental factor correlation [13] may compromise an appropriate response by *Cephalotus*, if water availability or its covariate is not well integrated as a cue modulating the phenotypic plasticity.

Supplementary Methods

Plant materials. Because it is impractical to obtain a large number of individuals (>3,000 plants) from wild populations of *Cephalotus follicularis*, which is currently listed as a "vulnerable" species on the IUCN Red List of Threatened Species [14], we used a vegetatively propagated axenic culture strain (i.e., *in vitro* plants), which we obtained previously from CZ Plants Nursery (Trebovice, Czech Republic) for the genome sequencing [15]. Although the geographical origin of this strain is unknown, a species misidentification is unlikely because the genus *Cephalotus* and the family Cephalotaceae are both monotypic (i.e., *C. follicularis* is the only extant species) [12]. As whole-genome sequences are already available [15], molecular genetic studies on the geographical differentiation of this species will reveal the origin of the cultivated strain we used. Throughout the manuscript, we discussed the results of our laboratory experiments by taking into account the meteorological similarities and differences in four representative localities that cover the distribution limits of *Cephalotus* (see "Meteorological data" and Fig. S2). The plants were maintained in polycarbonate containers (60 × 60 × 100 mm) containing half-strength Murashige and Skoog medium [16] supplemented with 3% sucrose, 1× Gamborg's vitamins, 0.1% 2-(N-morpholino)ethanesulfonic acid, 0.05% Plant Preservative Mixture (Plant Cell Technology), and 0.3% Phytagel (Fig. S10). The plants were vegetatively propagated by shoot cuttings, and therefore the plant materials used in this study were effectively clonal with little to no genetic variation. Newly propagated plants were supplied for experiments (*i.e.*, plants were not reused for multiple experiments).

Heterophylly of *Cephalotus*. *Cephalotus* produces two types of pitcher leaves: juvenile and adult pitcher leaves [12,17]. Young plants tend to bear juvenile pitchers that are later replaced by adult pitcher leaves. They differ in size, overall shape, and the presence of some features, including a toothed rim [12], but both of them are functional for prey trapping. This can be considered a leaf shape plasticity that occurs over the lifespan of plants (i.e., heteroblasty [18]) rather than that occurs in response to environmental cues (i.e., heterophylly) because the juvenile-to-adult transition is usually irreversible in its natural habitats [19]. In our culture conditions, only juvenile pitchers were produced, and therefore, the production of malformed leaves identified in this study (Fig. 1C–I) most

Deleted: The

Formatted: Font: Italic

Formatted: Font: Italic

Deleted: and therefore

Deleted: Fig. S9

Deleted: heteroblastic development

likely involve the disturbed developmental programs of flat leaves and juvenile pitcher leaves rather than that of adult pitcher leaves. Flat leaves exhibit heteroblastic changes too but it is not so pronounced as to be easily discernible. The heteroblastic state of flat leaves in our culture conditions are most likely juvenile because small plantlets were used in the experiments, but this is not conclusive.

Deleted: the heterophylly we refer to here is the phenotypic plasticity between flat leaves and juvenile pitcher leaves in *Cephalotus*.

Analysis of heterophylly. To examine plant phenotypes under controlled and constant environments, we used aseptic cultures for the experiments. Plants were precultured for months at 25°C in continuous light (24L0D). A preculture at 15°C did not substantially change the pattern of heterophyllous phenotypes in continuous light (Fig. S8). We also examined the effect of light intensity ranging 8 to 54 μ mol m^{-2} s^{-1} under continuous light. Because no substantial effect was observed (Fig. S9), the rest of the experiments were conducted under a light intensity of 20 to 40 μmol m^{-2} s^{-1}. To ensure uniform plant conditions at the onset of the experiments, we selected asexually produced shoots with one or two expanded leaves and transferred them to a new medium. Paraffin-embedded tissue sections of a typical shoot tip were prepared as described previously [20], and are illustrated in Fig. S11, where the youngest two leaf primordia do not show signs of flat- or pitcher-leaf morphogenesis. Either nine or 16 plants were allocated to each container. The plants were then grown for approximately 12 weeks, and subsequently, we recorded the morphology of the produced leaves and their phyllotactic positions in the main shoots. Leaves on axillary shoots were omitted. All experiments were conducted in growth chambers (MIR-150, MIR-153, MIR-154, and MLR-350 by PHC, CLE-405 by TOMY SEIKO, LP-1PHS by Nippon Medical & Chemical Instruments Co., Ltd., PK 520-LED by poly klima GmbH, and their equivalents) with controlled temperature and daylength (Fig. S10).

Deleted: Fig. S10

Deleted: Fig. S9

Heterophylly in different temperatures and photoperiods. Although the ambient temperature in the natural *Cephalotus* habitats fluctuates daily, we used constant temperatures that are within a range of seasonal fluctuations of daily mean temperature. When combined with an appropriate day length, the use of daily mean temperature reproduces a key component of the seasonal transcriptomes in *Arabidopsis halleri* subsp. *gemmifera* [21], suggesting that constant temperatures are a reasonable approximation of daily fluctuating temperatures (see Supplementary Text 1 for further

discussion). The effect of photoperiod was initially tested under short-day (8L16D) and long-day (16L8D) conditions, schemes commonly used for model plants. We also examined a range of naturally occurring photoperiods (10L14D, 12L12D, and 14L10D). In most experiments, 32 or 45 plants were examined for heterophylly (Table S3). We performed the experiments one to five times (Table S3) and presented the pooled data because consistent results were obtained among the individual experiments.

Leaf categorization. We used two schemes of leaf categories: binarized, in which malformed leaves were amalgamated to either flat or pitcher leaves (flat-like, F, C, H, M, and I; pitcher-like, L, D, and P; see Table S1), or fully categorized, in which all types of malformed leaves in Fig. 1 were recorded as is. The binarized scheme was used only in the experiments presented in Fig. 2B,C and Fig. S8, while the fully categorized scheme was used for all other experiments (Table S3).

Meteorological data. The daily measurements of temperature from 1907 to 2016 in a representative habitat of *Cephalotus* (35.02°S, 117.88°E, Albany, Australia) were retrieved from the website of Bureau of Meteorology (BOM) in the Australian Government (http://www.bom.gov.au/climate/data/). The Albany region has been recognized as a representative habitat because of abundant specimen records (Fig. S2) since its discovery within this region in the early 1800s [12]. Minute-by-minute temperature measurements at the Albany airport were also acquired from the BOM for the estimation of daily mean temperature. Other on-site meteorological data were purchased from meteoblue (http://meteoblue.com). The photoperiod was calculated using the python package 'PyEphem' (https://github.com/brandon-rhodes/pyephem). Because the geographic distribution of *Cephalotus* ranges over 400 km of the south-western coastal regions in Western Australia and Albany is close to its eastern limit [12], we also examined other locations including Yallingup, the historically recorded north-western limit or an outlier distribution [22] (33.69°S, 115.04°E) (Fig. S2). The seasonal changes of temperature and photoperiod in Yallingup were similar to those in Albany, with slightly higher temperatures (Fig. S2).

Estimation of daily mean temperature. The daily mean temperature was estimated with the metric called T_{sim}, which takes into account daily minimum/maximum temperatures,

two coefficients for daytime and nighttime temperature changes (C_D and C_N), and astronomical sunrise/sunset times [23]. We estimated the two coefficients using iterative grid searches using minute-by-minute temperature measurements at the Albany airport from 2012 to 2016 ($C_D = 0.978$; $C_N = 1.006$). The implementation is available on GitHub (https://github.com/kfuku52/averagers). Consistent with previous work [23], T_{sim} outperformed the average of the daily extremes in estimating the daily mean temperature at Albany (Fig. S12). A five-fold cross-validation yielded the root mean square error of 0.72°C.

Deleted: Fig. S11

Numerical simulation. Generalized linear models were fitted to meteorological data using the python package 'scipy' [24]. Correlated Gaussian and beta distributions were generated using the R package 'mvtnorm' [25].

Analysis of heterophylly data. In addition to the experiments in this study, we incorporated some data from previous work [15] (Tables S2–S3). Because the oldest one or two leaves were already mature at the onset of our experiments, we excluded the oldest two leaves from all data analyses except the plots in Fig. 2B,C.

Deleted: S2

Statistical analysis. All data were processed using python or R. Statistical analyses were performed using 'scipy'. The data were visualized using 'matplotlib' [26], 'seaborn' (https://github.com/mwaskom/seaborn), 'python-igraph' (https://igraph.org/python/), and 'ggplot2' [27]. Box plot elements of all figures are defined as follows: center line, median; box limits, upper and lower quartiles; whiskers, 1.5× interquartile range; points, outliers.

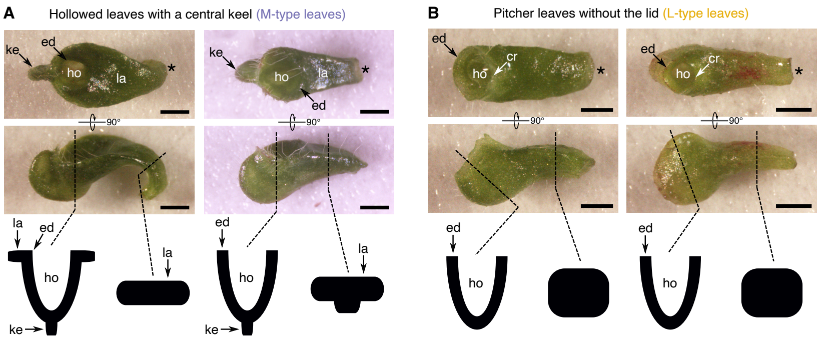

Fig. S1. Sectional representations of two categories of malformed leaves. (**A**) Hollowed leaves with a central keel. (**B**) Pitcher leaves without the lid. Bars indicate 1 mm. Asterisks indicate the position of the leaf base. Abbreviations: la, laminar portion; ho, hollow; ed, hollow edge; cr, protrusion in the cross zone. Although the laminar portion might look somewhat similar to lateral keels, they can be distinguished by their continuity with the true leaf margin, which is connected to the margin of the leaf base. The laminar portion is continuous, as seen in flat leaves, and lateral keels are not, as in pitcher leaves.

Deleted:

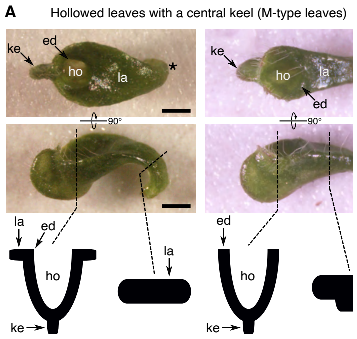

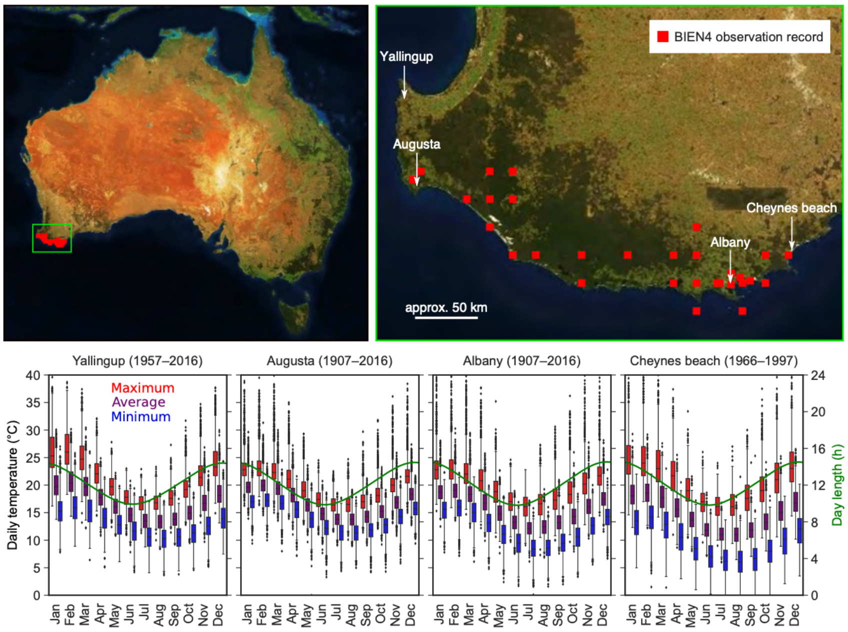

Fig. S2. Climates of the *Cephalotus follicularis* habitats. Specimen records of *Cephalotus follicularis* in the Botanical Information and Ecology Network version 4 (BIEN4; http://biendata.org/) are visualized on a map (upper). The region marked in green in the left panel is enlarged in the right panel. Seasonal fluctuation of the maximum, estimated average, and minimum temperatures, as well as day length, are shown for four locations (lower).

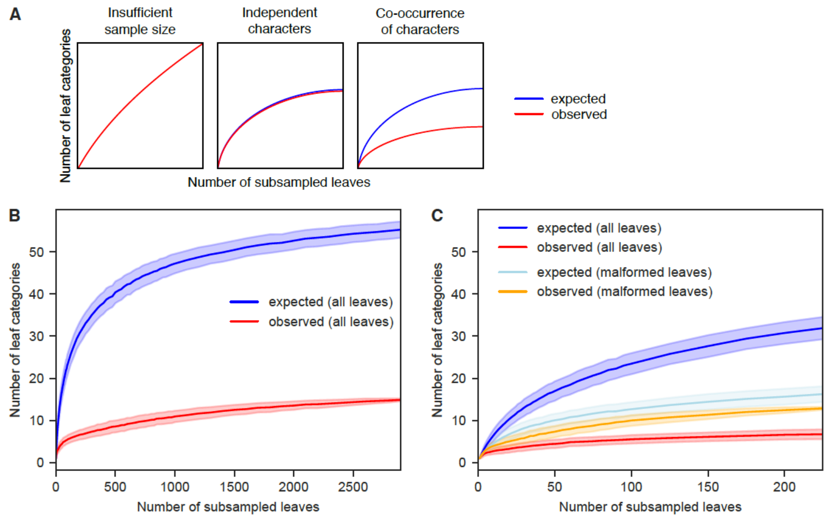

Fig. S3. A rarefaction curve analysis of the leaf categories. (**A**) Data interpretation. (**B**) The observation and expectation curves. The observed numbers of categories (red) were obtained by subsampling from 2,988 leaves in 700 individuals grown at 15.0°C, 17.5°C, 20.0°C, 22.5°C, or 25.0°C in continuous light. Expected numbers of leaf categories were calculated by randomizing the six morphological characters (la, ho, ke, lk, tr, and cr in Fig. 1), while retaining the observed trait frequencies. Category O was removed from this analysis. Category I was not observed in this dataset. The traits shown as "+/–" in Table S1 were treated as 0.5 counts per leaf in the estimation of trait frequency. The characters introduce a bias as a form of underestimating the observed counts. To report a conservative result, leaf categories with "+/–" characters were sub-divided into new categories with equal frequency covering all possible combinations of character states. As a result, H and M were divided into two categories each, and L and D were divided into four categories each. Standard deviation (shadow) was obtained from 1,000 iterations. The robustness of the expectation was validated in a stochastic simulation of leaf sampling under no trait correlation (Supplementary Data). (**C**) Analysis of 232 malformed leaves in comparison with the entire dataset. Flat leaves (F) and pitcher leaves (P) were rejected in the resampling for the non-correlated expectation curve of malformed leaves.

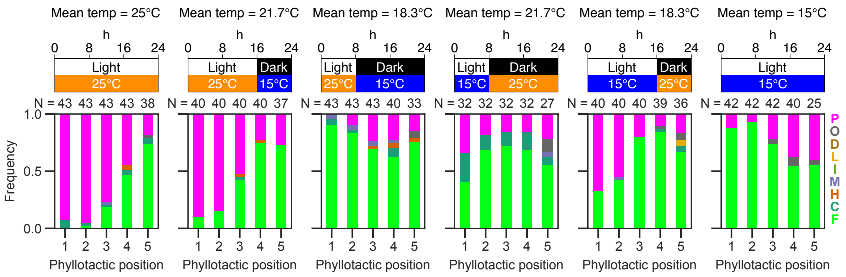

Fig. S4. The effect of daytime and nighttime temperatures. Plants were grown in different combinations of daytime/nighttime temperatures (15°C and 25°C) and photoperiods (8L16D, 16D8D, and 24L0D). Heterophyllous leaf production was recorded after a 12-week culture period. The numbers of observed leaves (N) and the regimes of temperature and photoperiod are indicated above the stacked bar plots. The colors of stacked bar plots and corresponding leaf category labels match those in Fig. 1.

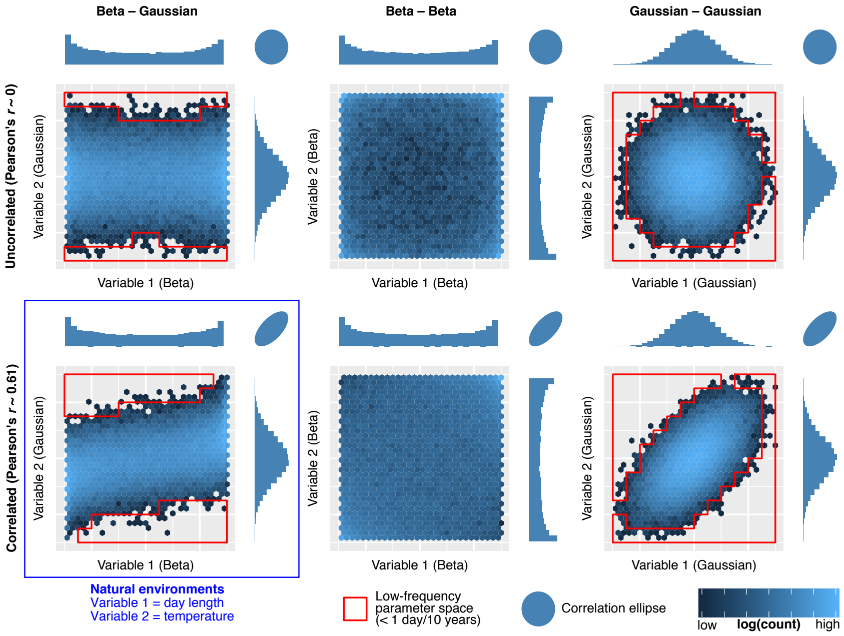

Fig. S5. Discordant combinations emerge in specific pairs of parameters. The natural parameter space (blue) was reproduced by fitting a beta distribution and a Gaussian distribution to day length and daily mean temperature, respectively, and by linking them with the observed value of Pearson's correlation coefficient. The discordance in their combinations (red) was diminished either by replacing the temperature-like Gaussian distribution with day length-like beta distribution (lower middle) or by uncoupling them (upper left). The covariation and the shape of distribution also influenced the shape and position of discordant combinations (upper right and lower right). In total, 36,500 data points were generated for each panel.

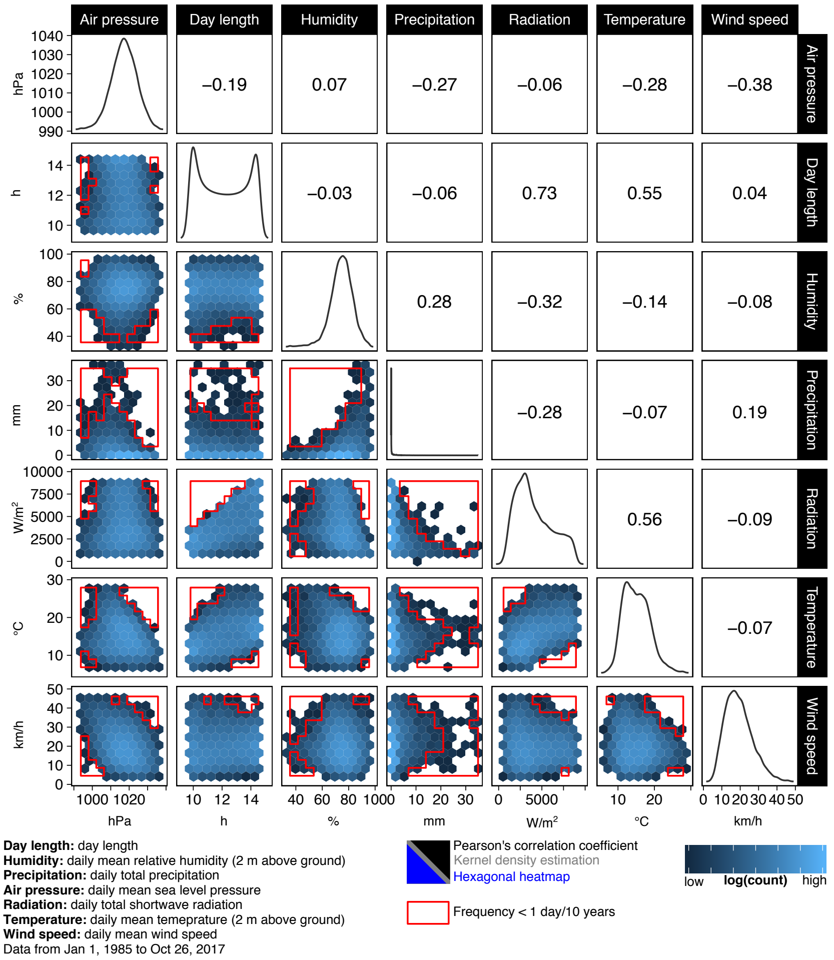

Fig. S6. Sparse parameter spaces are not limited to temperature and photoperiod. Although some pairs of environmental factors were almost completely orthogonal (e.g., day length vs. wind speed), many pairs showed discordant combinations in the parameter space (e.g., air pressure vs. temperature). The meteorological data in Albany were obtained from meteoblue (https://www.meteoblue.com/). For visualization purposes, the outlier values were clipped (lower 0.1% and upper 0.1%). Please note that some of the visualized sparse parameter spaces may be generated by the limitations of meteorological mechanisms (e.g., low humidity and high precipitation), even though such conditions can be created in a laboratory.

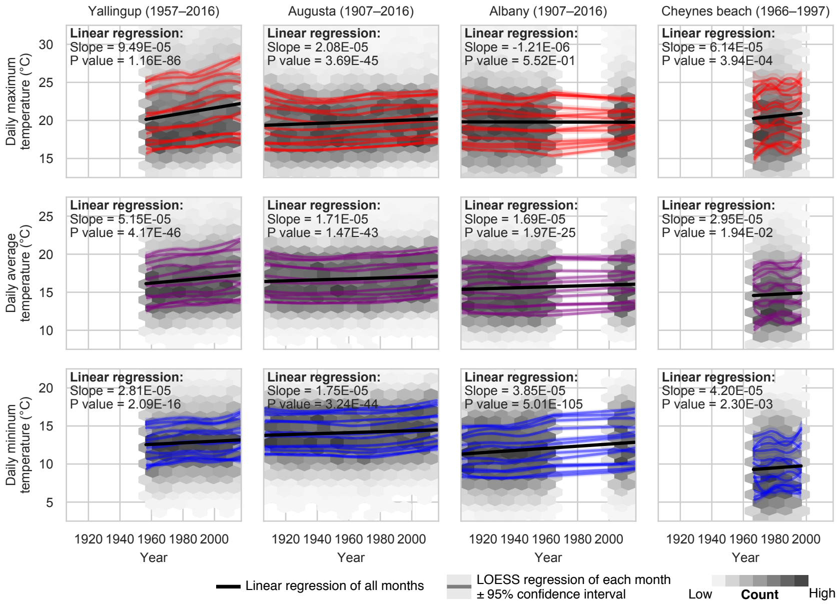

Fig. S7. Historical shifts of maximum, mean, and minimum temperatures in the habitats of *Cephalotus*. The mean temperatures are estimated values. Except for the daily maximum temperature in Albany, all of the estimated slopes are positive values, indicating a warming trend.

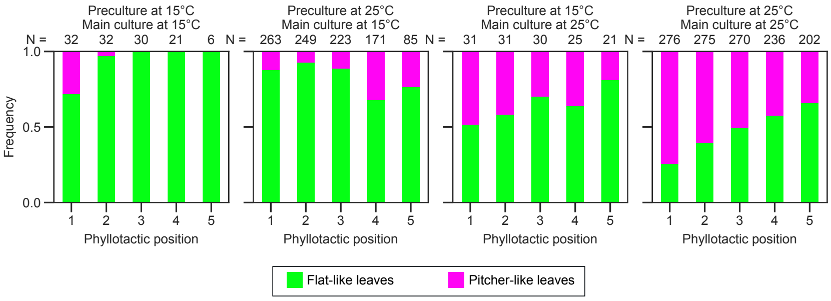

Fig. S8. The effect of preculture conditions. Plants were precultured either at 15°C or 25°C for months under continuous light. The heterophyllous leaf production was recorded after 12 weeks of the main culture at the specified temperature. All leaves at least in phyllotactic position 1 are expected to have been formed during the main culture, as shown in Fig. 2C. Note that malformed leaves were categorized into flat-like or pitcher-like in this analysis (i.e., “binarized” scheme, see Supplementary Methods and Table S1). The flat-like category represents categories F, C, H, M, and I, whereas the pitcher-like category includes categories P, L, and D. Although fully categorized data were not obtained, the proportion of malformed leaves was stably low. The numbers of observed leaves (N) are indicated above the stacked bar plots.

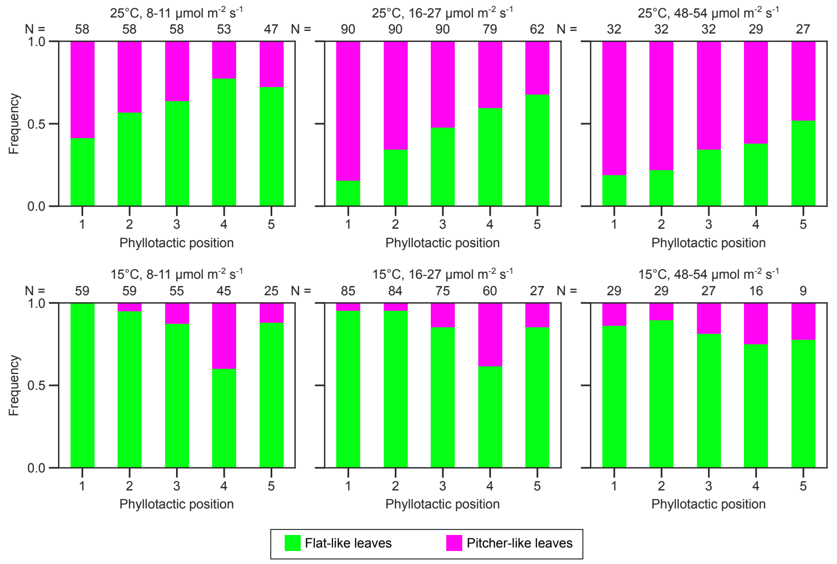

Fig. S9. The effect of light intensity. Heterophylly in different combinations of temperature and light intensity in continuous light (24L0D). The heterophyllous leaf production was recorded after 12 weeks of the main culture. Note that malformed leaves were categorized into flat-like or pitcher-like in this analysis (i.e., “binarized” scheme, see Supplementary Methods and Table S1). The flat-like category represents categories F, C, H, M, and I, whereas the pitcher-like category includes categories P, L, and D. Although fully categorized data were not obtained, the proportion of malformed leaves was stably low. The numbers of observed leaves (N) are indicated above the stacked bar plots.

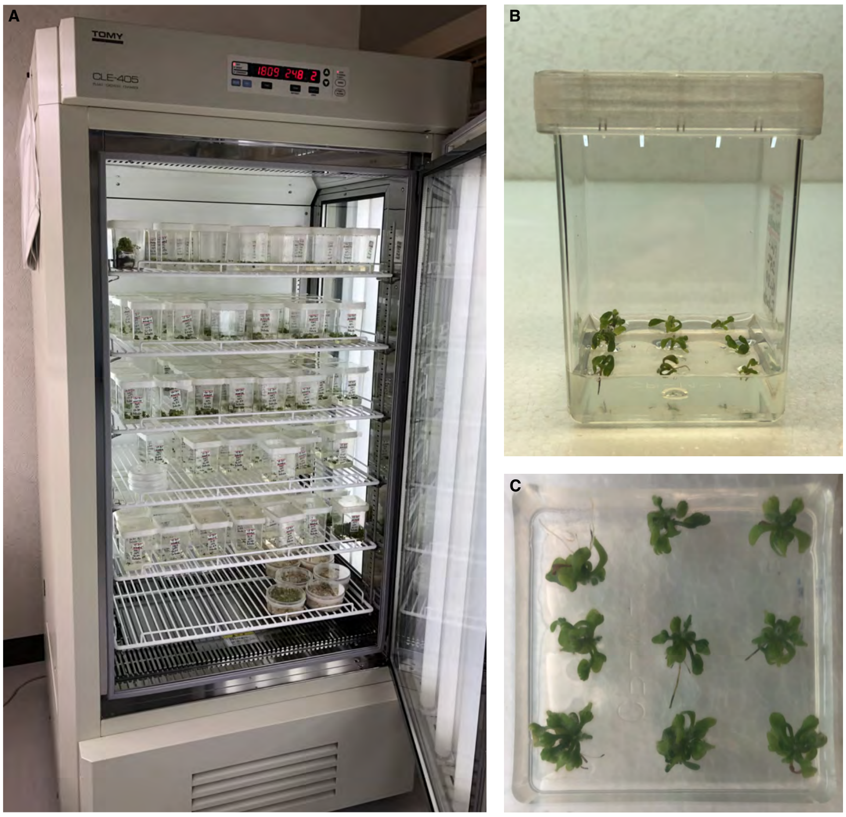

<u>Fig. S10</u>. Growth experiment in a climate chamber. (**A**) A front view of a growth chamber. (**B**) A lateral view of a plant container. (**C**) A top view of a plant container. In this container, nine *Cephalotus* plantlets were grown in a medium.

Deleted: **Fig. S9**

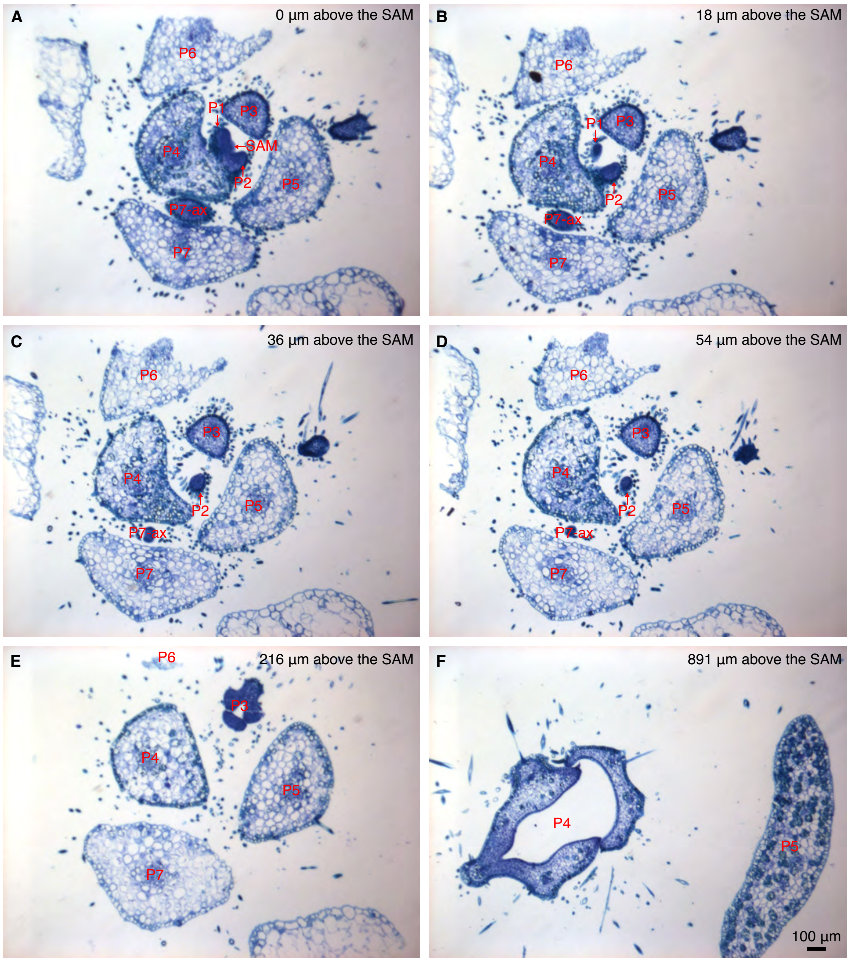

Fig. S11. Organization of the shoot tip in *Cephalotus*. (**A–F**) Transverse sections of the shoot apical meristem and leaf primordia. Nine-micrometer-thick paraffin-embedded serial sections were prepared from a plant grown at 25°C in continuous light, stained with 0.1% toluidine blue, and observed as described previously [20]. The transverse section at the level of the shoot apical meristem (**A**) shows the youngest visible leaf primordium (P1). In this shoot tip, P1 and P2 did not show clear signs of pitcher or flat leaf morphogenesis (**A–D**), whereas P3 and P4 were developing into pitcher leaves (**E,F**). P5 was developing into a flat leaf (**F**). Abbreviations: SAM, shoot apical meristem; P1–P7; leaf primordium numbers with the youngest represented by P1; P7-ax, the axillary shoot associated with P7. Two shoot tips were examined, and one sample is shown.

Deleted: Fig. S10

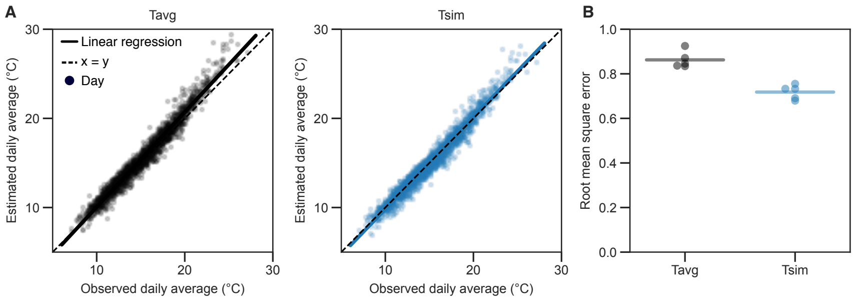

Fig. S12. The performance evaluation of two methods of daily mean temperature estimation. The estimated temperatures with T_{avg} and T_{sim} were related to the observed values (**A**). Points correspond to days. Their performance was evaluated with a five-fold cross-validation (**B**), in which observations from four years (2012 to 2016) were used as training data, and the remaining one year was reserved as the test data. Each point corresponds to an iteration in the cross-validation. Bars indicate the mean values of root mean square errors (RMSE).

Deleted: **Fig. S11**

Supplementary References

1. Burgie ES, Vierstra RD. 2014 Phytochromes: An atomic perspective on photoactivation and signaling. *Plant Cell* **26**, 4568–4583. (doi:10.1105/tpc.114.131623)

2. Jung J-H *et al.* 2016 Phytochromes function as thermosensors in *Arabidopsis*. *Science* **354**, 886–889. (doi:10.1126/science.aaf6005)

3. Legris M *et al.* 2016 Phytochrome B integrates light and temperature signals in *Arabidopsis*. *Science* **354**, 897–900. (doi:10.1126/science.aaf5656)

4. Paik I, Kathare PK, Kim J-I, Huq E. 2017 Expanding roles of PIFs in signal integration from multiple processes. *Mol. Plant* **10**, 1035–1046. (doi:10.1016/j.molp.2017.07.002)

5. Huang H *et al.* 2016 PCH1 integrates circadian and light-signaling pathways to control photoperiod-responsive growth in Arabidopsis. *eLife* **5**, e13292. (doi:10.7554/eLife.13292)

6. Nusinow DA, Helfer A, Hamilton EE, King JJ, Imaizumi T, Schultz TF, Farré EM, Kay SA. 2011 The ELF4–ELF3–LUX complex links the circadian clock to diurnal control of hypocotyl growth. *Nature* **475**, 398–402. (doi:10.1038/nature10182)

7. Salter MG, Franklin KA, Whitelam GC. 2003 Gating of the rapid shade-avoidance response by the circadian clock in plants. *Nature* **426**, 680–683. (doi:10.1038/nature02174)

8. Zhu J-Y, Oh E, Wang T, Wang Z-Y. 2016 TOC1–PIF4 interaction mediates the circadian gating of thermoresponsive growth in *Arabidopsis*. *Nat. Commun.* **7**, 1–10. (doi:10.1038/ncomms13692)

9. Seo PJ, Mas P. 2015 STRESSing the role of the plant circadian clock. *Trends Plant Sci.* **20**, 230–237. (doi:10.1016/j.tplants.2015.01.001)

10. Givnish TJ, Burkhardt EL, Happel RE, Weintraub JD. 1984 Carnivory in the bromeliad *Brocchinia reducta*, with a cost/benefit model for the general restriction of carnivorous plants to sunny, moist, nutrient-poor habitats. *Am. Nat.* **124**, 479–497. (doi:10.1086/284289)

11. Givnish TJ, Sparks WK, Hunter SJ, Pavlovič A. 2018 Why are carnivorous plants? Cost/benefit analysis, whole-plant growth, and the context-specific advantages of botanical carnivory. In *Carnivorous Plants: Physiology, ecology, and evolution*, pp. 232–255. Oxford University Press.

12. Cross A, Kalfas N, Nunn R, Conran John. 2019 Cephalotus—*the Albany Pitcher Plant*. England: Redfern Natural History Productions.

13. Givnish TJ. 2002 Ecological constraints on the evolution of plasticity in plants. *Evol. Ecol.* **16**, 213–242. (doi:10.1023/A:1019676410041)

14. Bourke G, Cross A, Nunn R, Kalfas N. 2020 *Cephalotus follicularis*. *IUCN Red List Threat. Species 2020* , e.T39635A19631881. (doi:10.2305/IUCN.UK.2020-1.RLTS.T39635A19631881.en)

15. Fukushima K *et al.* 2017 Genome of the pitcher plant *Cephalotus* reveals genetic changes associated with carnivory. *Nat. Ecol. Evol.* **1**, 0059. (doi:10.1038/s41559-016-0059)

16. Murashige T, Skoog F. 1962 A revised medium for rapid growth and bio assays with tobacco tissue cultures. *Physiol. Plant.* **15**, 473–497. (doi:10.1111/j.1399-3054.1962.tb08052.x)

17. McPherson S, Robinson A, Fleischmann A. 2009 *Pitcher plants of the old world*. Poole, Dorset, England: Redfern Natural History Productions.

18. Zotz G, Wilhelm K, Becker A. 2011 Heteroblasty—a review. *Bot. Rev.* **77**, 109–151. (doi:10.1007/s12229-010-9062-8)

19. Clarke SA. 1988 Seasonal growth and mortality of the pitchers of the Albany pitcher plant, *Cephalotus follicularis* Labill. *Aust. J. Bot.* **36**, 643–653. (doi:10.1071/BT9880643)

20. Fukushima K, Fujita H, Yamaguchi T, Kawaguchi M, Tsukaya H, Hasebe M. 2015 Oriented cell division shapes carnivorous pitcher leaves of *Sarracenia purpurea*. *Nat. Commun.* **6**, 6450. (doi:10.1038/ncomms7450)

21. Nagano AJ, Kawagoe T, Sugisaka J, Honjo MN, Iwayama K, Kudoh H. 2019 Annual transcriptome dynamics in natural environments reveals plant seasonal adaptation. *Nat. Plants* **5**, 74–83. (doi:10.1038/s41477-018-0338-z)

22. Willis JH. 1965 Historical notes on the W.A. pitcher plant *Cephalotus follicularis*. *West. Aust. Nat.* **10**, 1–7.

23. Dall'Amico M, Hornsteiner M. 2006 A simple method for estimating daily and monthly mean temperatures from daily minima and maxima. *Int. J. Climatol.* **26**, 1929–1936. (doi:10.1002/joc.1363)

24. Virtanen P *et al.* 2020 SciPy 1.0: fundamental algorithms for scientific computing in Python. *Nat. Methods* **17**, 261–272. (doi:10.1038/s41592-019-0686-2)

25. Genz A, Bretz F. 2009 *Computation of Multivariate Normal and* t *Probabilities*. Heidelberg: Springer-Verlag.

26. Hunter JD. 2007 Matplotlib: a 2D graphics environment. *Comput. Sci. Eng.* **9**, 90–95. (doi:10.1109/MCSE.2007.55)

27. Wickham H. 2009 *ggplot2: Elegant Graphics for Data Analysis*. New York: Springer-Verlag. (doi:10.1007/978-0-387-98141-3)
